# Retraction: The candidate tumor suppressor gene ECRG4 inhibits cancer cells migration and invasion in esophageal carcinoma

**DOI:** 10.1186/1756-9966-30-19

**Published:** 2011-02-16

**Authors:** Linwei Li, Chunpeng Zhang, Xiaoyan Li, ShihHsin Lu, Yun Zhou

**Affiliations:** 1 Oncology Department, Henan Provincial People's Hospital, Zhengzhou 450003, PR China; 2 State Key Laboratory of Molecular Oncology and Department of Etiology and Carcinogenesis, Cancer Institute & Hospital, Chinese Academy of Medical Sciences & Peking Union Medical College, Beijing 100021, PR China

## Retraction

The authors have retracted this article [1] as there was a large overlap with a previously published article in *International Journal of Cancer *[2]. Dr Lu ShihHsin, although listed as an author, was not aware of the publication in *Journal of Experimental & Clinical Cancer Research *and the grant reference number stated in the acknowledgements was incorrectly applied to this article.

## References

[B1] Li Linwei Zhang Chunpeng Li Xiaoyan Lu ShihHsin Zhou Yun The candidate tumor suppressor gene ECRG4 inhibits cancer cells migration and invasion in esophageal carcinoma Journal of Experimental & Clinical Cancer Research 2010 29 133 10.1186/1756-9966-29-133 PMC2958930 20937111

[B2] Li LW Yu XY Yang Y Zhanag CP Guo LP Lu SH Expression of esophageal cancer related gene 4 (ECRG4), a novel tumor suppressor gene, in esophageal cancer and its inhibitory effect on the tumor growth in vitro and in vivo Int J Cancer 2009 125 1505 1513 10.1002/ijc.24513 19521989

